# Simple separation of good quality bovine oocytes using a microfluidic device

**DOI:** 10.1038/s41598-018-32687-6

**Published:** 2018-09-24

**Authors:** Wataru Iwasaki, Kenichi Yamanaka, Daisuke Sugiyama, Yuki Teshima, Maria Portia Briones-Nagata, Masatoshi Maeki, Kenichi Yamashita, Masashi Takahashi, Masaya Miyazaki

**Affiliations:** 1 Advanced Manufacturing Research Institute, National Institute of Advanced Industrial Science and Technology, 807-1 Shuku-machi, Tosu, Saga 841-0052 Japan; 2 Department of Applied Biological Sciences, Faculty of Agriculture, Saga University, 1 Honjo, Saga, 840-8502 Japan; 3 Department of Molecular and Material Sciences, Interdisciplinary Graduate School of Engineering Science, Kyushu University, 6-1 Kasuga-koen, Kasuga, Fukuoka 816-8580 Japan; 4 Division of Applied Chemistry, Graduate School of Engineering, Hokkaido University, Kita 13, Nishi 8, Kita-ku, Sapporo, Hokkaido 060-8628 Japan; 5 Division of Agrobiology, Graduate School of Agriculture, Hokkaido University, Kita 9, Nishi 9, Kita-ku, Sapporo, Hokkaido 060-8589 Japan; 6 Department of Bioscience and Bioinformatics, School of Computer Science and Systems Engineering, Kyushu Institute of Technology, 680-4 Kawazu, Iizuka, Fukuoka, 820-8502 Japan; 7 Present Address: Q-may Laboratory Co., 1116 Miyake, Taketa, Oita 878-0007 Japan; 8 Present Address: Process Engineering Center, Organic Chemical Products Company, Daicel Corporation, 1239, Shinzaike, Aboshi-ku, Himeji, Hyogo 671-1281 Japan

## Abstract

We fabricated a simple microfluidic device for separation of bovine oocytes based on the oocyte quality to improve the conception rate of *in vitro* fertilization (IVF) by using good quality oocytes. The microfluidic device separates oocytes based on sedimentation rate differences in a sucrose buffer, which is dependent on oocyte quality. The microfluidic device has a 700 µm width, 1 mm height, and 10 mm long separation channel. Oocytes were injected from the upper half of the separation channel, and they flowed while sinking. The outlets of the separation channel were divided into upper and lower chambers. Good quality oocytes settled faster than poor quality oocytes in sucrose buffer; therefore, good quality oocytes were collected from the lower outlet. We performed IVF after the microfluidic separation of oocytes. The developmental rate to blastocysts of oocytes collected from the lower outlet was significantly higher than those collected from the upper outlet (36.0% vs. 14.1%). This result was comparable to that in the BCB staining method performed as a comparison method (BCB+ : 35.7%, BCB−: 15.4%). These findings indicate that our microfluidic device could be applied to oocyte separation and contribute to improvement of *in vitro* embryo production system.

## Introduction

Success of *in vitro* fertilization (IVF) depends highly on quality of oocytes^1^. Oocyte quality has been evaluated based on morphological observations and the degree of adhesion of the cumulus cells^2^. However, it is difficult to distinguish between good and fair quality oocytes, and results have been assigned based on the personal judgments of veterinarians or technicians. Therefore, it is necessary to develop a new sorting technique that can efficiently separate good and poor quality oocytes. Brilliant cresyl blue (BCB) staining is one oocyte selection method^3,4^. Oocytes stained by BCB show better quality in manipulation than unstained oocytes. Alm *et al*. demonstrated 34.1% embryonic development with stained oocytes by BCB against 3.9% of unstained oocytes^3^. However, this method uses visual observation and requires approximately 2 h to stain the oocytes. In addition, some recent studies do not recommend use of BCB stain for oocyte selection because of DNA damage^5^, chromosomal abnormality^6^, cleavage^7^, and apoptosis^8^ of oocytes caused by BCB staining^9^. In contrast, Yotsushima *et al*. reported that the speed of sedimentation of cumulus-oocyte complexes (COCs) and denuded oocytes in hypertonic solution correlated with the morphological quality of COCs^10^. This report indicated that the sedimentation method could be a useful method to objectively select good quality oocytes. With this method, non-toxic solutions should be used to separate oocytes. Sucrose solution is one of the ideal separation solution candidates because of its attributes, such as non-damaging to oocytes and ease in controlling the solution density. Liu *et al*. demonstrated that one hour exposure to 0.3 M sucrose in PBS containing 20% bovine fetal serum was harmless to the parthenogenetic development of bovine oocytes^11^. Therefore, we used a sucrose solution as a hypertonic solution.

Moreover, several newly developed high-throughput cell and particle sorting techniques have used microfluidic devices in the past few decades^12–15^. The microfluidic devices have various advantages, including easy fabrication and low cost. Nakahara *et al*. fabricated a robot-integrated microfluidic chip that can introduce and transport oocytes, measure their mechanical characteristics, and collect them automatically^16^. This method simplified evaluation of the mechanical characteristics of oocytes using an automated system; however, it lost the advantages of simple and low-cost fabrication of a microfluidic device because it requires a microelectromechanical system device and a control unit. Conversely, we have previously developed a density-based separation technique using a microfluidic device^17^. This method is based on sink-float separation of particles. Relative to the buffered solution, particles with a lower density floated while particles with a higher density sank. The density of the buffer was controlled by the sucrose concentration. In this study, we applied this previous technique to separate good quality oocytes based on differences in the sedimentation rate. Further, we validated the separation quality by estimating the developmental competence of separated oocytes after IVF.

## Results and Discussion

### Oocyte separation with microfluidic device at various flow rates

**Figure 1:**
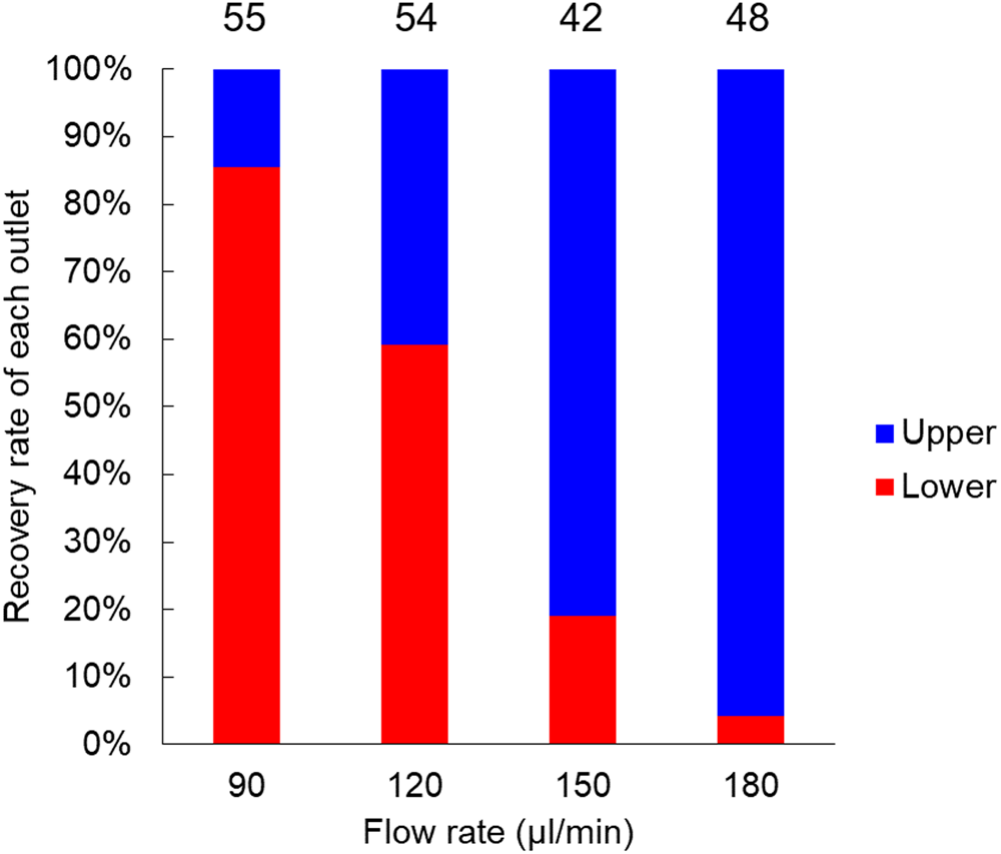
Recovery rate of oocytes from each outlet of the microfluidic device at various flow rates. The number above the bar represents the total number of oocytes at each flow rate.

To select the suitable flow rate, we conducted oocyte separation with the microfluidic device at various flow rates. Figure 1 shows the recovery rate from the upper and lower outlets at each flow rate. The number described above the bar indicates the total number of oocytes used for the separation at each flow rate. The number of oocytes used for these separations ranges from 42 to 55. When the flow rate was 90 µL/min, more than 80% oocytes were recovered from the lower outlet. In contrast, more than 80% oocytes were recovered from the upper outlet when the flow rate was greater than 150 µL/min. When the flow rate was 120 µL/min (SI movie), the ratio of oocytes recovered from the lower outlet to the upper outlet was 6:4, which is similar to that of the BCB+ group vs. BCB− group in the BCB test. Based on these results, we postulated that this flow rate was effective for oocyte separation. Therefore, subsequent experiments were performed at 120 µL/min.

### Cytoplasmic morphology of oocytes after oocyte separation with the microfluidic device

**Figure 2:**
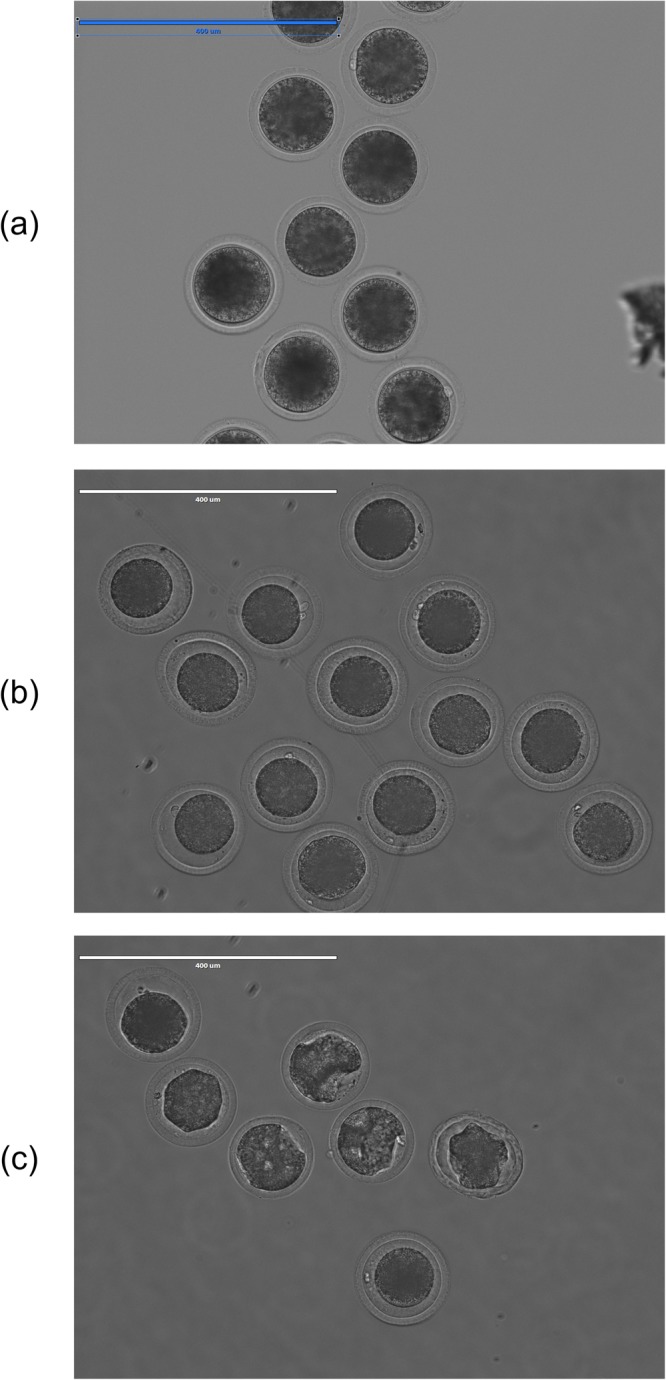
Microscopic images of oocytes before separation (**a**), and those recovered from the lower (**b**) and upper (**c**) outlets after separation using the microfluidic device. Scale bars represent 400 *μ*m.

The cytoplasmic shrinkage due to immersion into a hypertonic solution was observed during oocyte separation with the microfluidic device. Therefore, we compared cytoplasmic morphology of oocytes after shrinkage. Most oocytes recovered from upper outlet showed abnormal cytoplasm, while almost all oocytes recovered from the lower outlet maintained a normal, round shape as shown in Table 1 and Fig. 2. The oocytes recovered from the lower outlet had a significantly higher rate of normal cytoplasm compared with those from the upper outlet (95.5% vs. 24.5%, *P* < 0.01). These results suggest that the differences in oocyte cytoplasmic morphology exposed to hypertonic solution would affect the sedimentation rate. Although this cytoplasmic shrinkage recovered after immersion in TCM199 medium containing 5% FCS, morphological patterns of the oocyte cytoplasm in hypertonic solution were likely related to the distribution of cytoskeletons and organelles. It has been well established that eukaryotic cells require cytoskeletons to maintain their shape and for migration, growth, polarization, organelle movement, endocytosis/exocytosis, replication, and gene regulation. Previous studies showed that these distributions were related to oocyte maturation and oocyte competence^18–21^. Disruption of cytoskeletons caused developmental arrest in bovine oocytes^22^. Dang-Nguyen *et al*. reported differences in cytoplasmic shrinkage of porcine oocytes after treatment in a 0.2 M sucrose solution, and the misshaped oocytes showed abnormal organization of cytoskeletal components^19^. In addition, Stojkovic *et al*. reported that mitochondrial localization was different between morphologically good and poor quality oocytes^20^. Collectively, our results suggest that the microfluidic device enables separation of good quality oocytes in terms of morphology.

**Table 1:** Cytoplasmic morphology of oocytes after separation with a microfluidic device.

Outlet	Number of oocytes	Cytoplasmic morphology after separation		Normal rate (% ± SEM)
		Normal	Abnormal	
Upper	49	12	37	24.5 ± 2.8^a^
Lower	44	42	2	95.5 ± 4.4^b^

Cytoplasm morphology was observed after separation with a microfluidic device. Oocytes with a spherical cytoplasm were judged as normal, whereas oocytes with a non-spherical cytoplasm were judged as abnormal. The data were obtained from three replicates. Values with different letters differ significantly, P < 0.01.

### Developmental competence of oocytes separated by microfluidic device

To evaluate the developmental competence of oocytes separated with a microfluidic device, we prepared 360 oocytes apart from the previous separation test. These oocytes were separated using a microfluidic device at a 120 µL/min flow rate, because the 120 µL/min flow rate showed good balance in the number of recovered oocytes from each outlet. We performed IVF with the separated oocytes and assessed the rate of blastocyst formation on day 8. The number of oocytes separated by the microfluidic device, BCB staining, and conventional morphological methods by three different technicians and their developmental rate to blastocyst are shown in Table 2. After separation treatment, the number of collected oocytes from the upper (163) and lower outlet (197) was not different. However, blastocyst formation was significantly higher (*P* < 0.01) for oocytes collected from the lower outlet (36.0%) than from the upper outlet (14.1%). Notably, the developmental rate of oocytes from the lower outlet (36.0%) was similar to that by technician B (34.7%) and was significantly higher (*P* < 0.01) than that by technician A and C (20.6% and 28.3%, respectively).

**Table 2:** Results of *in vitro* development of separated oocytes by various methods followed by IVF.

Method		Number of oocytes	Number of blastocysts				Developmental rate to blastocyst (% ± SEM)
			Early blastocyst	Blastocyst expansion	Hatched blastocyst	Total	
Microfluidic	Upper	163	11	11	1	23	14.1 ± 1.3^a^
device	Lower	197	18	43	10	71	36.0 ± 1.2^b^
BCB	BCB−	143	12	9	1	22	15.4 ± 0.7^ac^
staining	BCB+	213	23	42	11	76	35.7 ± 1.2^b^
Conventional	A	412	31	47	7	85	20.6 ± 1.1^c^
morphological	B	375	38	86	6	130	34.7 ± 0.7^b^
evaluation	C	300	30	44	11	85	28.3 ± 1.5^d^

Oocytes collected from both upper and lower outlets were prepared for IVF and cultured, and their developmental rates to blastocyst stage were investigated on day 8. As a comparison method, BCB staining was performed to compare the separation capability between separation methods. The data were obtained from three replicates. Conventional morphological evaluation was performed by three different technicians (A, B, C) as a comparison method. COCs with a homogeneous spherical ooplasm and multilayered compact cumulus cells were selected by each technician and prepared for IVM and IVF. All technicians performed all procedures using the same protocols and materials. The data were obtained from six replicates. Values with different letters differ significantly, P < 0.01.

In the present study, the developmental rate to blastocyst can be estimated as 26.1%, even if separation was not conducted. However, taking into consideration the detrimental effects of poor quality embryos, the developmental rate without separation might have been lower than 26.1%. Group culture may promote embryo development via secretion and/or depletion of various factors in the media. Specifically, paracrine factors released by each embryo promote development of neighboring embryos in the group culture with a medium droplet^23^. Conversely, other factors derived from low-quality embryos may exert negative effects on the development of neighboring embryos^24^. Additionally, in the case of conventional morphological evaluation of COCs based on morphological observation^2^, developmental rates varied largely among technicians from 20.6% to 34.7%, even when the same protocols and materials were used for embryo production *in vitro*. It is assumed that this difference in the developmental rates could be due to variation among technicians in the selection criteria, as they determined which COCs to apply for IVM. In conventional IVF, COCs are only selected prior to IVM, and all selected COCs are applied to IVM followed by IVF. Thus, this selection would largely affect the success of IVF since developmental competence of embryos highly depends on oocyte quality^12^. Taken together, these findings suggest the significance of quality-based oocyte separation by a microfluidic device before IVF to obtain good developmental results, irrespective of the technician’s skill.

In the present study, to estimate the significance of oocyte separation with a microfluidic device, the BCB staining method, which is a well-established method, was also performed for comparison purposes^3,25,26^. The result of *in vitro* development of oocytes separated by BCB staining was comparable to the separation quality of microfluidic-sorted oocytes. The developmental rate to blastocyst of BCB+ and BCB− were 35.7% and 15.4%, respectively. This indicates that the microfluidic device has the same capability of oocyte separation as BCB staining. However, because oocyte separation by BCB staining takes more than 90 min, and selection by operators is based on oocyte coloration, this approach is time-consuming. In addition, some negative effects on DNA, chromosomes, and oocyte development have been reported^5–9^. In contrast, the microfluidic device is a simple and time-saving method that required no staining and selection by operators, and the 0.3 M sucrose buffer is harmless to oocytes^11^. These results demonstrated that oocyte separation with a microfluidic device is superior to BCB staining, offering fast separation and easy handling. However, oocyte separation using microfluidic device requires removal of cumulus cells before IVF. The removal of cumulus cells before IVF decreased the developmental rate of embryos in cattle as reported by a previous study^27^, but no detrimental effects of cumulus cell removal before IVF were observed in this study. Although the reason for this contradiction is unclear, it might be a possible obstacle for application of our microfluidic device for *in vitro* embryo production. Therefore, further studies will be needed to investigate the effects of cumulus cell removal on developmental competence of bovine embryos.

## Conclusions

In this study, we fabricated a microfluidic oocyte separation device based on differences in sedimentation rate, depending on oocyte quality. Further, we evaluated the embryonic developmental competence of the separated oocytes. As a result, oocytes collected from the lower outlet had higher developmental competence than those from the upper outlet. This result showed that the good quality oocytes had a higher sedimentation rate in the hypertonic solution, enabling efficient oocyte separation in the microfluidic device. We also observed differences in cytoplasmic morphology of oocytes immersed in a hypertonic solution between oocytes collected from the upper and lower outlets. It has been considered that differences in cytoplasmic morphology were likely caused by the distribution pattern of the cytoskeleton and organelles, which was related to developmental competence. In addition, we demonstrated the BCB staining method and the conventional morphological assessment by three different technicians for comparison and to emphasize the advantage of the microfluidic device in terms of reducing process time and eliminating the technician-specific performance factor, which could be highly associated with oocyte quality and can affect oocyte selection. The separation quality of the microfluidic device was comparable to separation by BCB staining. However, BCB staining usually takes more than 90 min. Conventional morphological evaluation also takes a huge amount of time to separate a large number of COCs. In contrast, a microfluidic device takes a few minutes to separate oocytes. In addition, evaluation of BCB staining and conventional methods depends on the subjective judgment of the technicians. For example, when BCB staining of some oocytes is uncertain due to darkened bovine ooplasm, it is difficult to divide all applied oocytes on the basis of their coloration^28^. In addition, we demonstrated that the developmental rates of embryos from IVF using oocytes selected by conventional methods by three technicians with different experiences varied statistically from 20.6% to 34.7%, even when the same protocols and materials were used for embryo production *in vitro*. As such, the microfluidic device is independent of technicians because the syringe pump conducts the separation automatically. The separation procedure using the microfluidic device is simpler compared to the BCB staining and the conventional methods. Thus, these results demonstrate the advantage of the present oocyte separation method over the conventional morphological method and BCB staining, offering advantages, such as simple and time-saving operation and good embryonic development without the subjective judgment of the operators.

## Methods

### Reagents and apparatus

Chemicals used in this study were the highest purity grade from Wako Pure Chemical Industries, Ltd. (Osaka, Japan) without further purification. Oocytes were flowed in phosphate buffered saline (PBS) with 0.05 wt% polyvinyl alcohol (PVA, 163–16355, Wako Pure Chemical Industries, Ltd.) as a sample solution. Further, a sucrose solution with the density of 1.11 g/ml was made with PBS containing 0.3 M sucrose and 0.05 wt% PVA. PVA was added to prevent agglomeration and adsorption of oocytes. In addition, 25 mM HEPES-buffered TCM199 (M199, 12340-030) and fetal calf serum (FCS) were purchased from Gibco (Grand Island, NY, USA) and used for the preparation of bovine oocytes. Follicle-stimulation hormone (FSH, AntrinR10, Kyoritsu Seiyaku, Tokyo, Japan) was also used for the preparation of bovine oocytes. Percoll solution (GE Healthcare, Little Chalfont, UK) and IVF100 solution (Research Institute for the Functional Peptides, Yamagata, Japan) were used for IVF experiments. Micromilling machine (Micro MC-2, PMT Corp., Fukuoka, Japan) was used to fabricate the mold of the microfluidic device. Syringe pump Model 198 (Harvard) and Model 100 (BAS, Tokyo, Japan) were used for injection of the sample solution and sucrose solution, respectively. Statistical analysis was conducted using the Ekuseru-Toukei 2015 (Social Survey Research Information Co., Ltd. Tokyo, Japan).

### Collection and *in vitro* maturation of bovine oocytes

Collection and *in vitro* maturation (IVM) of bovine oocytes were carried out as described previously^29^. Briefly, bovine ovaries were kept in a thermos bottle with sterile 0.15 M saline at 20 °C and were transported from a local slaughterhouse to the laboratory. COCs were collected by aspiration of ovarian antral follicles (2–6 mm in diameter), using a 10-mL syringe equipped with an 18-gauge needle. All COCs with multilayered compact cumulus cells were applied to IVM without selection based on ooplasm morphology for experiments using the microfluidic separation device and BCB staining method, whereas only COCs with a homogeneous spherical ooplasm and multilayered compact cumulus cells were applied to IVM for an experiment with the conventional IVF method. After washing three times with TCM199 supplemented with 5% FCS, approximately 50 COCs were matured in a 500 µL drop of TCM199 medium containing 5% FCS, 0.02 IU/mL of FSH, and 10 µg/mL gentamicin sulfate covered with mineral oil for 20 h at 38.5 °C in a humidified atmosphere of 5% CO_2_ in air. After *in vitro* maturation, COCs were randomly divided into each experimental group. Animal ethics committee approvals were not required as ovaries were obtained from cows that were slaughtered for commercial food production.

### Microfluidic separation device

**Figure 3:**
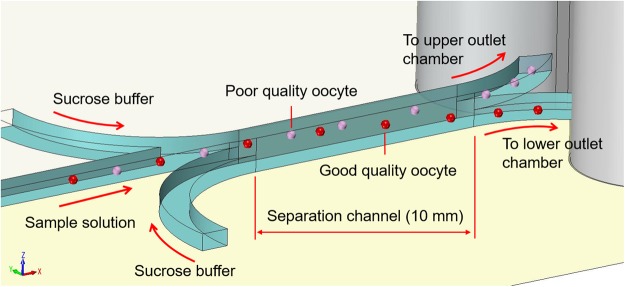
Schematic of oocytes separation in the microfluidic device.

**Figure 4:**
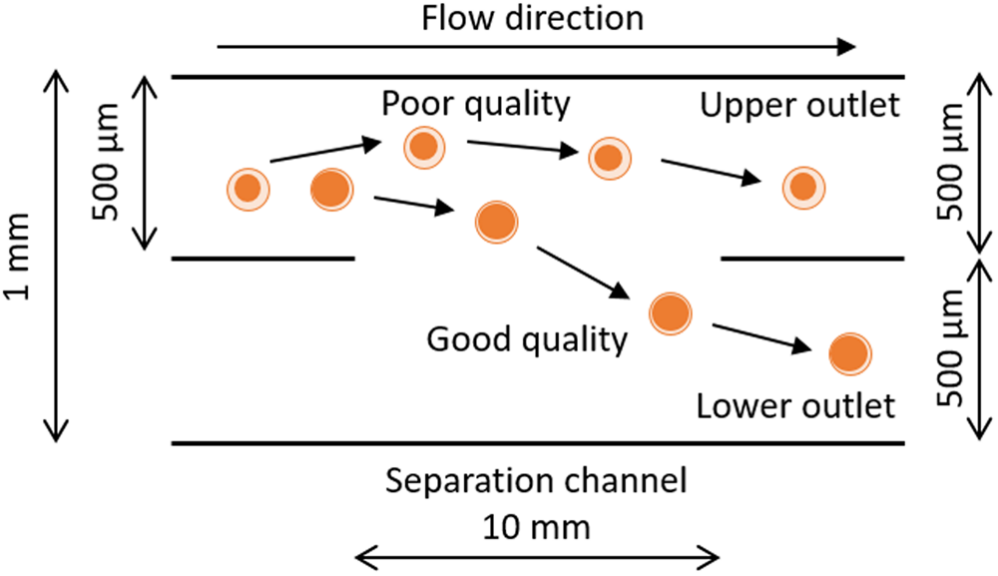
Schematic of the cross-sectional view of microfluidic separation devices.

The microfluidic separation device was designed based on a previously fabricated density-based particle separation device^17^. Figure 3 shows the concept of oocyte separation in a microfluidic device. The device has three inlets and two outlets. The sample solution was injected directly into the center inlet, forming a confluent stream with the sucrose buffer injected from the other two outside inlets. After forming the confluent stream, the oocytes flowed through the separation channel and gradually settled. The end of the separation channel was divided into upper and lower outlets and connected to reservoirs. The better and poorer quality oocytes were collected from the lower and upper outlet chambers, respectively. The separation results depended on the residence time of oocytes flowing through the separation channel, which could be controlled by the flow rate and length of the separation channel. In this study, the length of separation channel was fixed at 10 mm, and the residence time was controlled by the flow rate. Detailed design of the microfluidic device is shown in the supporting information (Fig. S1). In this study, we used the new design, in which the inlet for the sample solution was located at the upper side of the separation channel as shown in Fig. 4 because the oocytes were sorted according to differences in their sedimentation rate. The oocyte separation device was fabricated by a standard polydimethylsiloxane (PDMS) molding method, same as the previously developed particle separation device^17^. Briefly, two master polymethylmethacrlate (PMMA) molds of the upper plate and bottom plate were fabricated by a micromilling machine. The PDMS prepolymer was poured onto the PMMA master molds and cured at 70 °C for approximately 3 h, resulting in PDMS replicas of the upper and bottom plates. The microfluidic device was fabricated by assembling each PDMS replica after oxygen plasma pretreatment. The inlets for the sample and sucrose buffer, made of Teflon tubes, were connected to the microfluidic device and fixed into the microdevice using PDMS. The other side of the Teflon tube was connected to the gel loading pipette tip. The gel loading pipette tip was connected to a syringe pump (Model 100 BAS, Tokyo, Japan). The microchannel of the device was treated by immersing in 1 wt% PVA in deionized distilled water overnight at room temperature to prevent adsorption of oocytes on the inner wall of the device.

### Separation of bovine oocytes using microfluidic device

**Figure 5:**
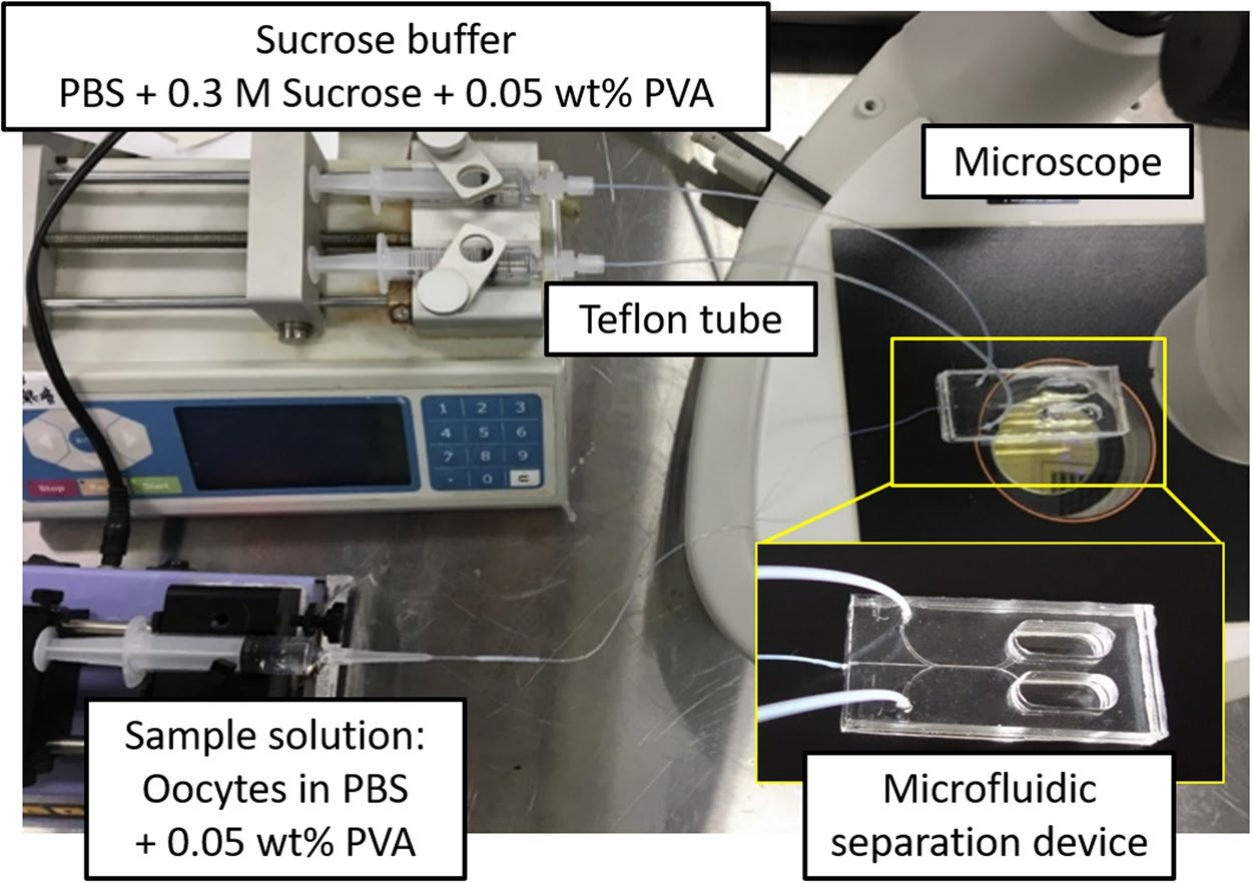
Experimental setup. The sample solution and sucrose buffer were loaded through the center inlet and two outside inlets of the microfluidic separation device, respectively, by syringe pumping. The circumstances of separation were monitored and recorded by video using the microscope.

The experimental setup for oocyte separation is shown in Fig. 5. A stereomicroscope (SMZ745T, Nikon, Tokyo, Japan) was used to observe the separated oocytes. The sample solution and sucrose buffer were loaded through the center inlet and two outside inlets, respectively, by syringe pumping. After IVM, expanded cumulus cells were removed by pipetting the COC in a solution of TCM199 containing 1 mg/mL of hyaluronidase. Approximately 20 denuded oocytes were collected from the culture medium and loaded approximately 20 µL of medium containing these oocytes into the broadened part of the gel loading pipet tip, in which the narrow tip was connected to the center inlet of the microfluidic device via a Teflon tube. Next, the broadened side of the gel loading pipet was connected to a syringe filled with the sample medium. Two syringes filled with sucrose solution were connected to two outside inlets of the microfluidic device. The denuded oocytes were sorted using the microfluidic device by flowing the sample solution and sucrose buffer through pumping. The residence time was controlled by flow rate. The separated oocytes were recovered from the upper and lower outlets and were applied to IVF.

### *In vitro* fertilization and culture

Frozen semen straws from a single Japanese Black bull were provided by Saga Prefectural Livestock Experiment Station and thawed by immersion of the straw in warm water (37 °C) for 20 sec. Animal ethics committee approvals were not required as semen were obtained from other governmental institutes. The spermatozoa were washed by centrifugation (800 *g* for 10 min) in 90% (v/v) Percoll solution. After removing the supernatant, the pellet was diluted with IVF100 solution, and further centrifugation was applied at 600 *g* for 5 min. The spermatozoa in pellet form were diluted with IVF100 to prepare the final sperm cell concentration at 5–10 × 10^6^/mL. After maturation, oocytes were washed three times with IVF100 and transferred into drops of sperm cells suspension (20 oocytes/100 µL drop). Fertilization was carried out for 6 h at 38.5 °C in a humidified atmosphere of 5% CO_2_ in the air. After removal of spermatozoa from the surface of the zona pellucida by gentle pipetting with a fine glass pipette, the putative zygotes were cultured in 50 µL micro-drops (20–30 zygotes/drop) of CR1aa containing 5% FCS at 38.5 °C in a humidified atmosphere of 5% O_2_, 5% CO_2_, and 90% N_2_ through day 8 (day 0 represented the day of insemination)^30^. Developmental competence was assessed by blastocyst rates on day 8.

### Experimental design

#### Experiment 1: Oocyte separation with microfluidic device at various flow rates

To determine the suitable flow rate for oocyte separation using the microfluidic device, we first performed oocyte separation at different flow rates and assessed the recovery rate from the upper and lower outlets. Flow rates at the separation channel ranged from 90 to 180 µL/min in increments of 30 µL/min. The residence times flowing in the separation channel with 10 mm length of each flow rate were 4.7 s, 3.5 s, 2.8 s, and 2.3 s, respectively.

#### Experiment 2: Cytoplasmic morphology of oocytes after oocyte separation with the microfluidic device

Since a sucrose buffer (hypertonic solution) was used for oocyte separation with the microfluidic device, oocyte cytoplasm shrunk during the separation process. In this experiment, we observed oocyte cytoplasm after separation with the microfluidic device and examined whether the shape of the shrunken cytoplasm differed between oocytes recovered from the upper and lower outlets. Oocytes with a spherical cytoplasm were judged as normal, whereas oocytes with a non-spherical cytoplasm were judged as abnormal.

#### Experiment 3: Developmental competence of oocytes separated by a microfluidic device

We evaluated separation capability of the microfluidic device in terms of developmental competence of the separated oocytes. Oocytes collected from both the upper and lower outlets were prepared for IVF and cultured, and their developmental competence was investigated. To compare the separation capability between separation methods, oocyte separation by BCB staining (see supporting information), which is a known reliable separation method for good quality oocytes, was performed as a comparison method^3,25,26^. In addition, to estimate the significance of oocyte separation using the microfluidic device, the developmental competence was investigated when oocytes were selected depending on their morphological characteristics by technicians, as a conventional method^2^. In this study, three technicians with different experiences conducted IVM and IVF using the same media and culture conditions. Technician A, B, and C have 1, 3, and 1 years’ experience of working as a technician, respectively. Each technician selected only COCs with a homogeneous spherical ooplasm and multilayered compact cumulus cells by microscope observation. Subsequently, they applied the selected COCs to IVM followed by IVF. Ovaries derived from a local slaughterhouse on the same day were also randomly divided and used by these technicians.

### Statistical analysis

The rate of normal cytoplasmic morphology between oocytes recovered from the upper and lower outlets after separation with a microfluidic device was analyzed by Student’s t-tests. Other data were analyzed using analysis of variance (ANOVA), followed by the Tukey-Kramer multiple comparison test. All percentage data were transformed to arc sine prior to statistical analysis. *P* < 0.05 was considered statistically significant.

## Electronic supplementary material

Supporting information

SI movie
