# Supplementary material for: Simple separation of good quality bovine oocytes using a microfluidic device
Source: Sci Rep. 2018 Sep 24;8:14273. doi: 10.1038/s41598-018-32687-6 (PMC6155318; doi:10.1038/s41598-018-32687-6)
Supplement: Supplementary file 1 — Supporting information [file 41598_2018_32687_MOESM1_ESM.docx]

**Supporting Information**

**Simple separation of good quality bovine oocytes by using a microfluidic device**

W. Iwasaki,^#a^ K. Yamanaka,^#b^ D. Sugiyama,^a†^ Y. Teshima,^c‡^ M. P. Briones-Nagata,^a^ M. Maeki,^c,d^ K. Yamashita,^a^ M. Takahashi^e^ and M. Miyazaki.^*a,c,d,f^

^a^*Advanced Manufacturing Research Institute, National Institute of Advanced Industrial Science and technology, 807-1 Shuku-machi, Tosu, Saga 841-0052, Japan.*

^b^*Department of Applied Biological Sciences, Faculty of Agriculture, Saga University, 1 Honjo, Saga, Saga 840-8502, Japan.*

^c^*Department of Molecular and Material Sciences, Interdisciplinary Graduate School of Engineering Science, Kyushu University, 6-1 Kasuga-koen, Kasuga, Fukuoka 816-8580, Japan.*

^d^*Division of Applied Chemistry, Faculty of Engineering, Hokkaido University, Kita 13, Nishi 8, Kita-ku, Sapporo, Hokkaido 060-8628, Japan. E-mail: m.miyazaki@eng.hokudai.ac.jp; Tel: +81-11-706-8188; Fax: +81-11-706-8188.*

^e^*Division of Agrobiology, Graduate School of Agriculture, Hokkaido University, Kita 9, Nishi 9, Kita-ku, Sapporo, Hokkaido 060-8589, Japan.*

*^f^Department of Bioscience and Bioinformatics, School of Computer Science and Systems Engineering, Kyushu Institute of Technology, 680-4 Kawazu, Iizuka, Fukuoka 820-8502, Japan*

^#^ These authors contributed equally to this work.

^†^ Present address: Q-may Laboratory Co., 1116 Miyake, Taketa, Oita 878-0007, Japan.

^‡^ Present address: Process Engineering Center, Organic Chemical Products Company, Daicel Corporation, 1239, Shinzaike, Aboshi-ku, Himeji, Hyogo 671-1281, Japan.

**Microfluidic Device**

**
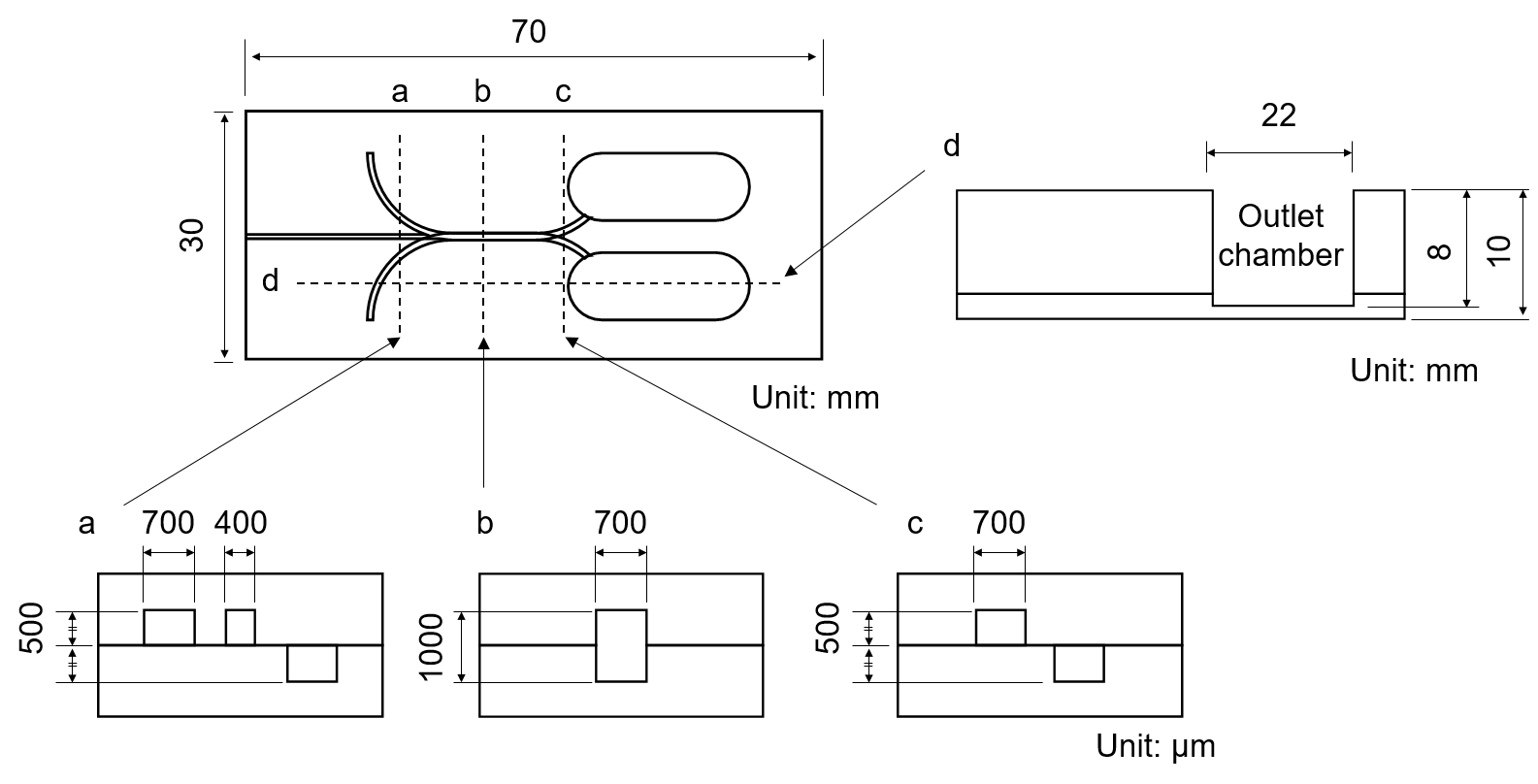
**

Fig. S1. Detailed dimension of microfluidic device used in this study.

**BCB staining**

After collection, COCs were washed in PBS containing 5% (v/v) FBS and were exposed to 26 mM of BCB (B-5388, Sigma) for 20 h at 38.5°C in a humidified atmosphere of 5% CO_2_ in air. Following BCB staining, COCs were washed twice in PBS containing 0.05 wt% PVA, and were divided into BCB+ group (blue colored cytoplasm) and BCB− group (colorless cytoplasm) depending on oocyte cytoplasm coloration. Each group was applied to IVM followed by IVF.

**Separation of Oocyte**

A movie was provided (SI movie).
